# Recent Advances and Perspectives in Terms of the Therapeutic Effects of Natural Products on Human Diseases

**DOI:** 10.3390/life16020245

**Published:** 2026-02-02

**Authors:** Seung Ho Lee

**Affiliations:** Department of Nano-Bioengineering, Incheon National University, 119 Academy-ro, Incheon 22012, Republic of Korea; seungho@inu.ac.kr; Tel.: +82-32-835-8269

## 1. Introduction

Natural agents have long been recognized as valuable resources to discover functional ingredients that can be developed into therapeutics to treat intractable and rare diseases. Although developing therapeutic agents derived from natural sources requires labor-intensive processes, including extensive screening, purification, and a rigorous evaluation of efficacy, natural agents offer distinct advantages over synthetically designed compounds, particularly in terms of reduced toxicity and fewer adverse side effects [1]. Moreover, natural agents often yield unexpected bioactive compounds with unique mechanisms of action, thereby opening up new therapeutic possibilities for diseases that remain difficult to treat using conventional approaches.

These advantages have contributed to a growing interest in natural agents within the field of therapeutic development. Indeed, a substantial proportion of currently approved therapeutics have been developed directly or indirectly from natural products [2]. In addition, numerous studies continue to reveal the novel therapeutic potential of natural agents derived from medicinal plants [3,4], marine algae [5,6], microorganisms [7,8], and functional foods [9,10].

The present Special Issue, “Therapeutic Effects of Natural Products on Human Diseases, 2nd Edition”, published in *Life*, comprises eight original research articles, three review articles, and one case report that collectively highlight recent advances in the therapeutic evaluation of natural products. This Editorial provides an overview of the key findings presented in this Special Issue and discusses their scientific significance, with particular emphasis on the diverse therapeutic effects of natural agents across a broad spectrum of human diseases.

## 2. Highlights from This Special Issue

In their original study, Jang et al. [11] present a systems biology-based framework to elucidate the therapeutic scope and underlying molecular mechanisms of National Health Insurance herbal prescriptions used in traditional Korean medicine. The authors demonstrate that network-predicted disease profiles show significant concordance with clinically validated indications for the majority of prescriptions. This study represents a methodological advancement by introducing a statistically validated and reproducible computational framework that extends beyond descriptive network analysis.

Kwon et al. [12] comprehensively summarize the therapeutic potential of *Ishige okamurae* Yendo as a natural multi-target agent for dementia management by integrating evidence from in vitro and in vivo models of Alzheimer’s disease, glutamate excitotoxicity, and bacterial-driven neuroinflammation. *I. okamurae* extract consistently ameliorates cognitive deficits and neuronal damage by modulating distinct yet interconnected molecular pathways. By synthesizing mechanistic evidence across multiple dementia-related disease models, this review reframes dementia therapy from a single-target approach toward a multi-pathway, systems-level strategy and positions *I. okamurae* as a promising prototype for natural multi-target therapeutics.

Wattanathorn et al. [13] addressed the cognitive-enhancing effects of a carotenoid-enriched functional congee containing dried tomato powder in healthy elderly volunteers through a randomized, double-blind, placebo-controlled clinical study. The authors report that daily consumption of the functional congee significantly improves cognitive processing and working memory, which are associated with reductions in serum α-synuclein and monoamine oxidase levels, as well as a selective increase in *Lactobacillus* spp. in the gut microbiota. These findings support the concept that functional foods may contribute to dementia prevention strategies by modulating multiple interconnected biological pathways.

In a double-blind, placebo-controlled clinical trial, Park et al. [14] evaluate the immunomodulatory effects of *Lacticaseibacillus rhamnosus* LM1019 on natural killer (NK) cell activity in generally healthy adults. The authors demonstrate that LM1019 supplementation significantly enhances NK cell activity in a defined adult subset. This study provides clinically meaningful evidence that probiotic-induced immune enhancement depends on host baseline characteristics rather than being uniform across populations and highlights the potential of *L. rhamnosus* LM1019 as a safe, non-pharmacological strategy to counteract early immunosenescence.

In this Special Issue, several studies highlight the anti-inflammatory potential of natural agents. Min et al. [15] present a narrative review examining the role of plant-based dietary patterns in preventing and managing inflammatory skin diseases. In an original research article, Lee et al. [16] demonstrate the anti-inflammatory efficacy of *Hyeonggaeyeongyo-tang* (HGYGT), a traditional herbal prescription widely used for inflammatory otorhinolaryngological disorders. Molecular evidence for the anti-inflammatory effects of HGYGT is provided using both in vitro and in vivo inflammatory models, together with standardized chemical profiling of the formulation. By offering mechanistic insight and translationally relevant evidence, this study advances the understanding of how multi-component herbal formulations such as HGYGT exert targeted immunomodulatory actions. Han et al. [17] investigated the anti-inflammatory activity and human skin safety of *Mosla japonica*, a traditional Eastern medicinal herb. The authors elucidate the molecular mechanisms underlying the anti-inflammatory effects of *Mosla japonica* extract and propose clinically applicable doses based on direct human safety validation. Collectively, these findings provide essential evidence supporting the potential use of *Mosla japonica* extract as a functional therapeutic and skincare ingredient.

In their review, Singh et al. [18] explore the role of anaerobic spore-forming *Clostridium* species as innovative anti-cancer agents, with a particular focus on their unique ability to selectively target hypoxic regions within solid tumors. The authors summarize recent advances in *Clostridium*-based cancer therapies and highlight how combination approaches with chemotherapy, radiotherapy, and immunotherapy may overcome the intrinsic limitations of conventional cancer treatments in hypoxic tumor microenvironments. Collectively, this review outlines a forward-looking roadmap for clinically translating *Clostridium*-based therapies as promising complementary modalities capable of targeting therapy-resistant hypoxic tumor niches.

A compelling exosome-based neuroprotective strategy for Parkinson’s disease (PD) was provided by Chiang et al. [19]. The authors demonstrate that resveratrol-enhanced human neural stem cell-derived exosomes (RES-hNSCs-Exos) effectively mitigate 1-methyl-4-phenylpyridinium (MPP^+^)-induced neurotoxicity in SH-SY5Y cells. These neuroprotective effects are mediated through improvements in mitochondrial function, enhanced ATP production and mitochondrial biogenesis, and reductions in oxidative stress. Collectively, these findings advance the understanding of exosome-mediated neuroprotection and suggest that resveratrol-enhanced exosome production may represent a promising platform for developing exosome-based therapeutics for PD and related neurodegenerative disorders.

In a case report, Cho et al. [20] describe the successful management of suspected nonatypical endometrial hyperplasia using modified *Sihogyeji-tang* monotherapy, with objective evaluation by serial transabdominal ultrasonography. Notably, normalizing endometrial thickness was achieved without progesterone therapy, highlighting the potential of herbal formulations to modulate abnormal endometrial proliferation while simultaneously improving systemic and menstrual symptoms and maintaining endometrial thickness within the normal physiological range. This study provides valuable clinical insight and supports the rationale for future controlled studies investigating nonhormonal, patient-centered approaches to estrogen-dependent gynecological disorders.

Sheir et al. [21] demonstrate the nephroprotective effects of aged black garlic (ABG) extract in a sodium benzoate-induced chronic kidney injury model in albino rats. The authors show that ABG extract markedly attenuates renal damage by mitigating oxidative stress and inflammatory responses. This study advances the current understanding of how functional foods may protect against environmentally and diet-induced chronic kidney disease. Importantly, the findings provide scientific evidence supporting the potential of aged black garlic as a promising nutraceutical candidate for kidney health preservation.

Faris [22] reports the anti-cancer efficacy of *Verbascum ponticum* (Stef.) flower extract in non-small-cell lung carcinoma. The extract markedly suppresses the proliferation and viability of A549 cells by disrupting mitochondrial membrane potential and promoting the translocation of apoptosis-inducing factor (AIF) from mitochondria to the nucleus, thereby inducing caspase-independent apoptotic cell death. Importantly, *V. ponticum* extract exhibits selective cytotoxicity toward cancer cells, with minimal effects on normal bronchial epithelial cells, highlighting its potential therapeutic relevance.

## 3. Final Reflections

This Special Issue highlights recent advances in the development of diverse and innovative therapeutic candidates derived from natural agents. Collectively, the findings presented herein reinforce the therapeutic potential of natural products and support their value as fundamental scientific resources for developing safe and effective therapeutics.
